# Analysis of the virulence potential, ability to form biofilms and susceptibility to bacteriocins of *Staphylococcus aureus* strains isolated from livestock and wildlife

**DOI:** 10.2478/jvetres-2026-0005

**Published:** 2026-02-05

**Authors:** Aleksandra Trościańczyk, Aneta Nowakiewicz, Andrea Lauková, Marcelina Osińska, Agata Hahaj-Siembida

**Affiliations:** Sub-Department of Veterinary Microbiology, University of Life Sciences in Lublin, 20-950 Lublin, Poland; Institute of Animal Physiology, Centre of Biosciences of the Slovak Academy of Sciences, 84104 Bratislava, Slovak Republic

**Keywords:** antimicrobial resistance, bacteriocins, biofilm, lactic acid bacteria, virulence genes

## Abstract

**Introduction:**

The aims of the study were to analyse the ability of *Staphylococcus aureus* isolated from livestock and wildlife to produce biofilm, the presence of virulence genes and their distribution of within individual sequence types among these strains, and to assess the activity of bacteriocins isolated from *Enterococcus* and *Lactococcus* spp. against methicillin-resistant *S. aureus* (MRSA).

**Material and Methods:**

Biofilm formation was assessed using the crystal violet assay. The occurrence of 29 virulence genes was examined by PCR. The analysis of the activity of 13 bacteriocins was carried out using the agar spot test.

**Results:**

All the *S. aureus* strains had the ability to form biofilm. Nineteen virulence genes were detected, the most abundant of which were *icaA, icaR, clfA* and *eno* (all 100%), *icaD* (99%), *icaB* (85%), *fnbpA* (93%), *cidA* (87%), *ebps* (83%), *sdrC* (77%) and *sdrE* (76%). Correlations between the occurrence of *clfB* and *fib* and strong biofilm formation as well as between the occurrence of S*eC, hla, hlb* and *fnbpB* and methicillin resistance were observed (P-value < 0.05). A high degree of heterogeneity in virulence profiles was observed in a host-dependent pattern. Among MRSA belonging mainly to sequence type 398, virulence profiles XII and XIII dominated, including several of the noted genes. The bacteriocins produced by *L. lactis, E. asini* and *E. saccharolyticus* had a growth-inhibiting effect on MRSA, nisin having the highest activity.

**Conclusion:**

The presence of several virulence genes in the same *S. aureus* strain with demonstrable drug resistance and its ability to form biofilms indicate livestock and wildlife as potential reservoirs of pathogens dangerous to public health. The activity of some bacteriocins against MRSA could offer a promising alternative to antibiotic therapy.

## Introduction

Although *S. aureus* is a Gram-positive bacterium that normally colonises the skin and mucous membranes of the nasopharynx in humans and animals as a commensal, it can also be responsible for numerous superficial skin infections including abscesses, boils and ulcers; invasive diseases like pneumonia, cardiovascular infections and sepsis; and food poisoning (49). The frequent carriage of *S. aureus* in humans and various animal species additionally favours the phenomenon of host-switching and transmission of pathogenic strains from animals to humans and *vice versa*, posing a threat to public health.

One of the important features of *S. aureus* that plays a key role in the pathogenesis of the diseases it causes is its ability to produce a biofilm, which is a structure that allows bacteria to survive and evade the immune response. A mature biofilm consists of bacterial colonies organised in an extracellular matrix as well as extracellular polymeric substances, such as polysaccharides, nucleic acids and proteins (36). Biofilm formation is a complex phenomenon involving the simultaneous expression of many genes, and is one of the features of staphylococci involved in the pathogenesis of some diseases like endocarditis, arthritis or catheter-related bacteraemia (14). The first step in biofilm formation is adhesion, where the main role is played by microbial surface components recognising adhesive matrix molecule (MSCRAMM) adhesins (17). These include such factors as fibronectin-binding proteins A and B (FnbpA and FnbpB), fibrinogen-binding protein (Fib), clumping factors A and B (ClfA and ClfB), collagen-binding protein (Cna), *Staphylococcus* elastin binding protein (EbpS), laminin binding protein (Eno) and serine-aspartate repeat proteins (Sdr) (36). An important factor involved in biofilm formation is also biofilm-associated protein (Bap), which promotes adhesion to host cells and abiotic surfaces (36). Subsequently, polysaccharide intercellular adhesin (PIA) is synthesised, the formation of which is encoded by the *icaADBC* operon. This operon contains genes encoding the intracellular adhesion N-acetylglucosaminyl transferase enzyme synthesising oligomers (IcaA), the intracellular adhesion chaperone protein folding IcaA (IcaD), the intracellular adhesion deacetylase involved in PIA maturation (IcaB) and the intracellular adhesion protein transporting the PIA polymer across the cell membrane (IcaC). Another agent responsible for biofilm formation is the murein hydrolase regulator (CidA), which, through cell lysis and the release of environmental DNA, increases adherence on the one hand and contributes to the formation of the biofilm matrix on the other hand (40). Many other virulence factors are involved in the pathogenesis of *S. aureus*-induced diseases. One of the most important is the staphylococcal family of superantigens, which includes enterotoxins (SEs), classified as pyrogenic exotoxins causing food poisoning or release of cytokines and, consequently, toxic shock (this in the specific case of the SE staphylococcal toxic shock syndrome toxin (TSST) (4, 45). In turn, exfoliative toxins (ETs) are responsible for staphylococcal scalded skin syndrome and bullous impetigo in infants, young children and adults (38). Other important virulence agents of *S. aureus* that cause the formation of pores in the cell membranes of host cells are haemolysins and leukotoxins: Panton–Valentine leukocidin (PVL), encoded by the Panton–Valentine leukocidin S and F gene complex, and Leukocidin ED (LukED), encoded by the leukocidin E and D gene complex. These factors, through invasion, degradation of host cells and evasion of the body’s defence forces, participate in the pathogenesis of such diseases as pneumonia, sepsis, dermonecrotic skin infection, peritonitis, infection of the central nervous system and endocarditis (2).

Treatment of infections caused by *S. aureus* is one of the most serious challenges in medicine because of the growing phenomenon of drug resistance. In addition to natural and acquired mechanisms of drug resistance, biofilm formation is an equally common cause of therapeutic failure (33). All these features merit *S. aureus*’ classification among the ESKAPE (*Enterococcus faecium, S. aureus, Klebsiella pneumoniae, Acinetobacter baumannii, Pseudomonas aeruginosa* and *Enterobacter* spp.) pathogens capable of “escaping” conventionally used antimicrobial drugs (31). Methicillin-resistant *Staphylococcus aureus* (MRSA) is a particularly serious problem in hospitals worldwide and in veterinary medicine. Conventionally, MRSA strains are divided into hospital-associated, community-associated and livestock-associated subsets, but the boundaries of this division are becoming increasingly blurred because of the interpenetration of host environments (8). Methicillin resistance in *Staphylococcus* means lack of susceptibility to all drugs from the β-lactam group and frequently coexists with multidrug resistance (MDR) (8).

The progressive increase in the phenomenon of drug resistance among bacteria, especially those posing a serious threat to human and animal health like *S. aureus*, indicates the need to develop new strategies for combatting microorganisms. So far, bacteriophages, vaccinations or natural products of plant origin have been the alternatives to antibiotics and chemotherapeutics (18). Bacteriocins may also be a promising option with which to replace conventional methods of treating bacterial infections. These substances are mostly small ribosomally synthesised peptides with antimicrobial activity produced by bacteria. Their advantage over drugs is that they have a narrow spectrum of activity, often limited to a defined group of pathogens, and they spare beneficial biota (9). Bacteriocins show structural diversity even within bacteria belonging to the same species, which makes them a research subject in which much is left to learn. While commercial preparations based on the activity of bacteriocins are widely used in the food industry, bacteriocin use in veterinary medicine is still at the experimental stage (19). This is despite their high therapeutic potential, which has been demonstrated in successful treatment of bovine mastitis caused by *S. aureus* and of infections with Gram-positive and -negative bacteria in pigs and also implied by their positive effect on the intestinal flora in horses and rabbits (19). One of the main groups of microorganisms producing bacteriocins are lactic-acid bacteria belonging to the *Enterococcus* and *Lactococcus* genera (9). *Enterococcus* spp. bacteria are commonly found in the digestive tracts of humans and animals. They also play a significant role as starter cultures in fermented foods and have also been used as probiotics (10). *Lactococcus* species are acid bacteria widely used in dairy processing for the production of fermented food. Because they are classified as “generally recognised as safe”, they can be used both as live microorganisms (probiotics) and as their metabolites or cell fragments (postbiotics) (10).

The aims of the study in respect of *S. aureus* characterisation were to analyse the ability of strains isolated from animals to produce biofilm, detect the presence of virulence genes and determine the distribution of virulence gene profiles within individual sequence types (STs). The aim as concerns bacteriocins was to assess the activity of these peptides isolated from *Enterococcus* spp. and *Lactococcus* spp. against MRSA.

## Material and Methods

### Study material

The material for the research included 99 *S. aureus* strains isolated from the nasal cavities of livestock and wildlife. A total of 51 strains originated from pigs, 36 had colonised cows and 12 were from wild deer (*Capreolus capreolus* and *Cervus elaphus*) in Poland. The study material had partially been described previously (48). Isolation of bacteria was performed as part of monitoring studies on *S. aureus* carriage among animals on 14 farms without clinical symptoms. The material collected from wild deer came from animals culled in accordance with the regulations of the Hunting Law Act of 20 May 2020 (Journal of Laws 2020 No. 67, items 148, 695 and 875.) and the Regulation of the Minister of Climate and Environment of 29 June 2022. The resistance of the strains to antibacterial substances was assessed using the disc-diffusion method for 14 drugs (penicillin, cefoxitime, erythromycin, clindamycin, ciprofloxacin, tetracycline, enrofloxacin, chloramphenicol, gentamicin, nitrofurantoin, rifampin, linezolid, quinupristin-dalfopristin and trimethoprim-sulfamethoxazole) and the broth microdilution method for vancomycin according to the Clinical and Laboratory Standards Institute (6). The strains were tested for the presence of genes encoding resistance to methicillin (*mecA* and *mecC*), penicillin (*blaZ)*, vancomycin (*vanA* and *vanB*), macrolides (*ermA, ermC* and *msrA*), tetracycline (*tetL, tetK* and *tetM*), chloramphenicol (*cat (pC221), cat (pC194)* and *cat (pC223)*) and aminoglycosides (*aac(6')-Ie-aph(2")-Ia* and *aph(3')-IIIa*) (47). Multidrug-resistant and vancomycin-intermediate *S. aureus* (VISA) were subjected to multilocus sequence typing (MLST) (47). A list of the characteristics of the strains used for the study is presented in Supplementary Table S1.

### Assessment of biofilm production

All strains (n = 99) were assessed for biofilm formation. For this purpose, a previously described procedure using crystal violet was used with a modification (5). Briefly, overnight cultures were inoculated into trypticase soy broth (TSB) containing 0.25% glucose. Culture density was adjusted to approximately 0.5 McFarland standard (1–2×108 CFU/mL). Each broth culture was diluted 1 : 100 in TSB, and 200 μL of each culture was distributed to three wells of a 96-well microtitre plate and incubated for 24 h at 37°C. Bacteria-free TSB was the negative control. After the incubation, nonadherent cells were removed and the wells were washed three times with sterile phosphate-buffered saline. After drying, the bacterial cells were stained with 200 μL of 2% (w/v) crystal violet for 15 min and rinsed with water. Dry plates were flooded with 95% ethyl alcohol. The optical density (OD) was measured at 550 nm in a Model 680 plate reader (Bio-Rad, Hercules, CA, USA). The result was taken as the average of three readings. The strains were categorised according to their OD compared to the OD of the negative control (ODc) as follows: the strain was considered as biofilm negative if its OD ≤ OD_c_, the strain was classified as weakly adherent if the ODc < OD ≤ (2× OD_c_), it was moderately adherent if (2× ODc) < OD ≤ (4× OD_c_) and strongly adherent if (4× OD_c_) < OD.

### Virulence gene detection

All the *S. aureus* strains (n = 99) were evaluated for the presence of virulence genes and genes related to biofilm production. Bacterial DNA was isolated using the Gram Plus & Yeast Genomic DNA Purification Kit (EURx, Gdańsk, Poland) according to the manufacturer’s instructions. Isolates were tested for the virulence factors and encoding genes given in Table 1. The primers used in the study and the PCR reaction conditions were described in previously published articles (3, 4, 7, 20, 22, 28, 30, 32, 34, 41, 46).

**Table 1.** Virulence factors and encoding genes tested for in Polish livestock-and wildlife-originating *Staphylococcus aureus* strains

Virulence factor	Encoding gene(s)
*Staphylococcus* enterotoxins A–E	*SeA, SeB, SeC, SeD*, and *SeE*
Toxic shock syndrome toxin	*Tst*
Exfoliative toxin	*Et*
Panton–Valentine leukocidin components S and F	*lukS*-PV-*lukF*-PV
Leukocidin ED	*lukE* and *lukD*
Biofilm-associated protein	*bap*
α-haemolysin	*hla*
β-haemolysin	*hlb*
Polysaccharide intercellular adhesin	*icaABCD* and *icaR*
Clumping factors A and B	*clfA* and *clfB*
Fibronectin-binding proteins A and B	*fnbpA* and *fnbpB*
Murein hydrolase regulator	*cidA*
Collagen-binding protein	*cna*
*Staphylococcus* elastin-binding protein	*ebpS*
Laminin-binding protein	*eno*
Serine-aspartate repeat proteins	*sdrC, sdrD* and *sdrE*

### Assessment of the antimicrobial activity of bacteriocins

Because isolating bacteriocins is difficult and methicillin resistance among *S. aureus* is the key public health issue where bacteriocins may hold promise, the activity of bacteriocins was only tested against MRSA strains (n = 40; 39 isolated from pigs and 1 isolated from a cow). The research was carried out using 13 bacteriocins isolated from *E. faecium* (n = 6), *E. mundtii* (n = 1), *E. asini* (n = 1), *E. saccharolyticus* (n = 1) and *Lactococcus lactis* (n = 4) kindly provided by Dr Andrea Lauková from the Slovak Academy of Sciences. The activity of bacteriocins produced by *E. asini* and *L. lactis* against some *S. aureus* strains was partially described previously (24, 27). The complete list of bacteriocins used in the study is provided in Table 2. The analysis of the activity of bacteriocin-like inhibitory substances was carried out using the agar spot test as described previously (11). Briefly, brain heart infusion agar (BHIA; BD Difco, Sparks, MD, USA) was overlaid with 0.7% BHIA supplemented with 200 μL of 18-h culture of MRSA strains (with optical density at 600 nm of 1.0; 12.0 × 10^8^ CFU/mL). Two-fold dilutions of each bacteriocin (10 μL) were applied to the plate and incubated at 37°C for 18 h; next, growth inhibition zones were read. The activities of bacteriocins were expressed in arbitrary units per millilitre (AU/mL) as the reciprocal of the highest dilution of bacteriocins showing complete inhibition of the growth of the tested strain.

**Table 2.** Bacteriocins used in this study

Bacteriocin	Producer bacteria	Source of isolation
Ent412	*Enterococcus faecium* EF412	faecal strain from a Slovak warmblood horse
EntM/AL41	*E. faecium* AL41/CCM8558	environment
Ent7420	*E. faecium* EF2019/CCM7420	rabbit faeces
EntA(P)/EK 13	*E. faecium* EK13=CCM7419	environment
Ent55	*E. faecium* EF55	gastrointestinal tract of a chicken
Ent4231	*E. faecium* CCM4231	rumen of a calf
EM41/3	*E. mundtii* EM41/3	faeces of a horse of the Norik of Muran breed
Ent. as.^1^	*E. asini* EAs 1/11D27	inner part of the auricle mucosa of a mare of the Norik of Muran breed
Ent. sacch.	*E. saccharolyticus* Es 3/11D27	inner part of the auricle mucosa of a mare of the Norik of Muran breed
L.l MK 2/8^1^	*L. lactis*	raw goat milk
L.l MK 2/2^1^	*L. lactis*	raw goat milk
L.l MK 2/7^1^	*L. lactis*	raw goat milk
nisin	*L. lactis* (from Nisaplin)	commercial strain

1 – the activity of bacteriocins against some *S. aureus* strains was partially described previously (24, 27)

### Statistical analysis

Statistical analysis was performed using Statistica 13.1 (Dell, Round Rock, TX, USA). The Mann–Whitney U test (P-value < 0.05) was used to compare the occurrence of virulence genes in *S. aureus* isolated from different host species. The correlation between the occurrence of virulence genes, biofilm formation and methicillin resistance was determined using the Spearman’s rank correlation coefficient (P-value < 0.05). The correlation was assessed based on r_s_ values, where 0–0.19 was a very weak correlation, 0.20–0.39 a weak one, 0.40–0.59 a moderate one, 0.60–0.79 signified strong correlation and 0.80–1.0 was evidence of a very strong correlation.

## Results

**Fig. 1.**
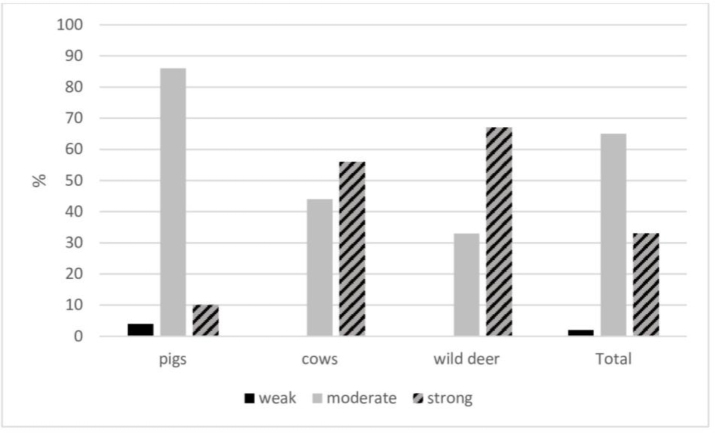
Biofilm production capacity of *Staphylococcus aureus* strains isolated from different hosts

All the tested *S. aureus* strains showed the ability to produce biofilm (Fig. 1). Most strains (65%) were moderate biofilm producers, while only 2% had a weak biofilm production capacity. The bacteria’s ability to produce biofilm depended on the host species from which the strains were isolated: most *S. aureus* strains isolated from cows and wildlife were strong biofilm producers (56% and 67%, respectively), whereas most strains isolated from pigs (86%) were moderate producers.

Of the 29 virulence genes screened for, 19 were found: *SeC, lukED, hla, hlb, icaA, icaB, icaD, icaR, clfA, clfB, fnbpA, fnbpB, cidA, fib, cna, ebpS, eno, sdrC* and *sdrE* (Table 2). No *SeA, SeB, SeD, SeE, tst, et, lukS*-PV*- lukF*-PV, *bap, icaC* or *sdrD* genes were detected among the *S. aureus* strains. All the strains were characterised by the presence of the *icaA, icaR, clfA* and *eno* genes. High-percentage presences of *icaD* (99%), *icaB* (85%), *fnbpA* (93%), *cidA* (87%), *ebpS* (83%), *sdrC* (77%) and *sdrE* (76%) were also found. The occurrence of some genes in *S. aureus* varied with the host species, statistically significantly so (P-value < 0.05) for *SeC, hla, hlb, clfB, fnbpB, fib* and *sdrC* (Table 3).

**Table 3.** Occurrence of virulence genes in Polish livestock- and wildlife-originating *Staphylococcus aureus* strains

	Virulence gene (n/%)																		
	SeC*	*lukED*	hla*	hlb*	*icaA*	*icaB*	*icaD*	*icaR*	*clfA*	clfB*	*fnbpA*	fnbpB*	*cidA*	fib*	*cna*	*ebpS*	*eno*	sdrC*	*sdrE*
Pigs (n=51)	36/71	1/2	39/76	39/76	51/100	36/71	50/98	51/100	51/100		44/86	38/75	38/75	14/27	40/78	36/71	51/100	39/76	36/71
Cows (n=36)		1/3	1/3	1/3	36/100	36/100	36/100	36/100	36/100	36/100	36/100	3/8	36/100	35/97	18/50	34/94	36/100	34/94	29/81
Wild deer (n=12)					12/100	12/100	12/100	12/100	12/100	12/100	12/100	2/17	12/100	12/100	9/75	12/100	12/100	3/25	10/83
Total (n=99)	36/36	2/2	40/40	40/40	99/100	84/85	98/99	99/100	99/100	48/48	92/93	43/43	86/87	61/62	67/68	82/83	99/100	76/77	75/76

1 *SeC* – gene C encoding *Staphylococcus* enterotoxin; *lukED* – genes E and D encoding leukocidins; *hla* – gene a encoding haemolysin; *hlb* – gene β encoding haemolysin; *icaA* – gene A encoding the intracellular adhesion N-acetylglucosaminyl transferase enzyme synthesising oligomers; *icaB* – gene B encoding the intracellular adhesion deacetylase involved in polysaccharide intercellular adhesin maturation; *icaD* – gene D encoding the intracellular adhesion chaperone protein folding IcaA; *icaR* – gene R encoding the intracellular adhesion transcriptional repressor of the ica operon; *clfA* – gene A encoding clumping factor; *clfB* – gene B encoding clumping factor; *fnbpA* – gene A encoding fibronectin-binding protein; *fnbpB* – gene B encoding fibronectin-binding protein; *cidA* – gene A encoding the murein hydrolase regulator; *fib* – gene encoding fibrinogen-binding protein; *cna* – gene encoding collagen-binding protein; *ebpS* – gene encoding *Staphylococcus* elastin-binding protein; *sdrC* – gene C encoding serine-aspartate repeat protein; *sdrE* – gene E encoding serine-aspartate repeat protein.

* – statistically significant differences in occurrence by host species (Mann–Whitney U test (P-value < 0.05))

The statistical analysis showed a moderate correlation between the occurrence of virulence genes and strong biofilm formation only for the *clfB* and *fib* genes (Table 4). These two virulence genes were strongly negatively correlated with methicillin resistance; however, the *SeC, hla, hlb* and *fnbpB* genes occurred in a very strong correlation with methicillin resistance. A very strong correlation was also observed between the occurrence of the *SeC* gene and the genes encoding haemolysins (*hla* and *hlb*) and *fnbpB* as well as between the occurrences of the *icaB, cidA* and *ebpS* genes (Table 4).

**Table 4.** Spearman’s rank correlation coefficient in the context of *Staphylococcus aureus* characteristics

	Biofilm	MRSA	*SeC*	*lukED*	*hla*	*hlb*	*icaB*	*icaD*	*clfB*	*fnbpA*	*fnbpB*	*cidA*	*fib*	*cna*	*ebpS*	*sdrC*	*sdrE*
Biofilm	-	–0.36*	–0.35*	0.05	–0.36*	–0.35*	0.24*	0.07	0.51*	0.19	–0.32*	0.27*	0.43*	–0.24*	0.21*	0.08	0.01
MRSA	–0.36*	-	0.92*	0.03	0.96*	0.94*	0.18	–0.12	–0.76*	0.07	0.81*	0.14	–0.75*	0.57*	0.21*	0.31*	0.32*
*SeC*	–0.35*	0.92*	-	–0.11	0.92*	0.89*	0.32*	0.08	–0.73*	0.21*	0.86*	0.29*	–0.70*	0.52*	0.34*	0.42*	0.43*
*lukED*	0.05	0.03	–0.11	-	0.17	0.18	–0.14	–0.70*	0.01	–0.24*	–0.13	–0.16	–0.03	0.10	–0.31*	–0.26*	–0.09
*hla*	–0.36*	0.96*	0.92*	0.17	-	0.98*	0.18	–0.12	–0.76*	0.07	0.81*	0.14	–0.70*	0.57*	0.16	0.26*	0.32*
*hlb*	–0.35*	0.94*	0.89*	0.18	0.98*	-	–0.17	–0.12	–0.74*	0.06	0.79*	0.13	–0.68*	0.56*	0.15	0.25*	0.31*
*icaB*	0.24*	0.18	0.32*	–0.14	0.18	–0.17	-	0.24*	0.41*	0.65*	0.26*	0.92*	0.07	0.37*	0.93*	0.57*	0.75*
*icaD*	0.07	–0.12	0.08	–0.70*	–0.12	–0.12	0.24*	-	0.10	0.37*	0.09	0.26*	0.13	–0.07	0.22*	0.18	0.18
*clfB*	0.51*	–0.76*	–0.73*	0.01	–0.76*	–0.74*	0.41*	0.10	-	0.27*	–0.65*	0.38*	0.72*	–0.24*	0.33*	0.01	0.12
*fnbpA*	0.19	0.07	0.21*	–0.24*	0.07	0.06	0.65*	0.37*	0.27*	-	0.16	0.71*	0.35*	0.15	0.60*	0.50*	0.49*
*fnbpB*	–0.32*	0.81*	0.86*	–0.13	0.81*	0.79*	0.26*	0.09	–0.65*	0.16	-	0.22*	–0.61*	0.34*	0.29*	0.38*	0.21*
*cidA*	0.27*	0.14	0.29*	–0.16	0.14	0.13	0.92*	0.26*	0.38*	0.71*	0.22*	-	0.12	0.30*	0.85*	0.56*	0.69*
*fib*	0.43*	–0.75*	–0.70*	–0.03	–0.70*	–0.68*	0.07	0.13	0.72*	0.35*	–0.61*	0.12	-	–0.37*	0.03	–0.09	–0.11
*cna*	–0.24*	0.57*	0.52*	0.10	0.57*	0.56*	0.37*	–0.07	–0.24*	0.15	0.34*	0.30*	–0.37*	-	0.31*	0.03	0.52*
*ebpS*	0.21*	0.21*	0.34*	–0.31*	0.16	0.15	0.93*	0.22*	0.33*	0.60*	0.29*	0.85*	0.03	0.31*	-	0.64*	0.74*
*sdrC*	0.08	0.31*	0.42*	–0.26*	0.26*	0.25*	0.57*	0.18	0.01	0.50*	0.38*	0.56*	–0.09	0.03	0.64*	-	0.47*
*sdrE*	0.01	0.32*	0.43*	–0.09	0.32*	0.31*	0.75*	0.18	0.12	0.49*	0.21*	0.69*	–0.11	0.52*	0.74*	0.47*	-

1 MRSA – methicillin-resistant *Staphylococcus aureus*; *SeC* – gene C encoding *Staphylococcus* enterotoxin; *lukED* – genes E and D encoding leukocidins; *hla* – gene a encoding haemolysin; *hlb* – gene β encoding haemolysin; *icaB* – gene B encoding the intracellular adhesion deacetylase involved in polysaccharide intercellular adhesin maturation; *icaD* – gene D encoding the intracellular adhesion chaperone protein folding IcaA; *clfB* – gene B encoding clumping factor; *fnbpA* – gene A encoding fibronectin-binding protein; *fnbpB* – gene B encoding fibronectin-binding protein; *cidA* – gene A encoding the murein hydrolase regulator; *fib* – gene encoding fibrinogen-binding protein; *cna* – gene encoding collagen-binding protein; *ebpS* – gene encoding *Staphylococcus* elastin-binding protein; *sdrC* – gene C encoding serine-aspartate repeat protein; *sdrE* – gene E encoding serine-aspartate repeat protein. Green indicates a very strong correlation and blue indicates a strong correlation

Twenty-two different virulence gene profiles were found among the isolated *S. aureus* strains, and they are detailed in Table 5. Most strains showed the simultaneous presence of several virulence genes, 16 genes being in profile XII, the most common at 30%; 14 genes being in profile I, a 15% proportion; 17 genes making up profile XIII, which 6% of strains had; and 15 genes comprising profile IX, the profile of 1% of multi-plevirulence-gene strains. The observed virulence gene profiles of *S. aureus* were all unique to one host group except profile IV, which was found in strains from cows and wild deer (Table 5). The most common profile among the isolates from pigs was XII (59%), among isolates from cows it was I (42%), and among isolates from wild deer it was VIII (67%). The virulence profiles detected among the strains isolated from pigs, cows and wild deer respectively comprised 5–17, 11–14 and 12–15 virulence genes (Table 5).

**Table 5.** Virulence gene profiles of *Staphylococcus aureus* strains (n/%)

Profile	Gene content	Pigs (n=51)	Cows (n=36)	Wild deer (n=12)	Total (n=99)
I	*icaA, icaB, icaD, icaR, clfA, clfB, fnbpA, cidA, fib, cna, ebpS, eno, sdrC, sdrE*		15/42		15/15
II	*icaA, icaB, icaD, icaR, clfA, clfB, fnbpA, cidA, fib, ebpS, eno, sdrC*		3/8		3/3
III	*icaA, icaB, icaD, icaR, clfA, clfB, fnbpA, fnbpB, cidA, fib, ebpS, eno, sdrC*		3/8		3/3
IV	*icaA, icaB, icaD, icaR, clfA, clfB, fnbpA, cidA, fib, ebpS, eno, sdrC, sdrE*		12/33	1/8	13/13
V	*icaA, icaB, icaD, icaR, clfA, clfB, fnbpA, cidA, fib, cna, eno*		1/3		1/1
VI	*lukED, hla, hlb, icaA, icaB, icaD, icaR, clfA, clfB, fnbpA, cidA, fib, cna, eno*		1/3		1/1
VII	*icaA, icaB, icaD, icaR, clfA, clfB, fnbpA, cidA, cna, ebpS, eno, sdrC, sdrE*		1/3		1/1
VIII	*icaA, icaB, icaD, icaR, clfA, clfB, fnbpA, cidA, fib, cna, ebpS, eno, sdrE*			8/67	8/8
IX	*icaA, icaB, icaD, icaR, clfA, clfB, fnbpA, fnbpB, cidA, fib, cna, ebpS, eno, sdrC, sdrE*			1/8	1/1
X	*icaA, iaB, icaD, icaR, clfA, clfB, fnbpA, fnbpB, cidA, fib, cna, ebpS, eno, sdrC*			1/8	1/1
XI	*icaA, icaB, icaD, icaR, clfA, clfB, fnbpA, cidA, fib, cna, ebpS, eno*			1/8	1/1
XII	*SeC, hla, hlb, icaA, icaB, icaD, icaR, clfA, fnbpA, fnbpB, cidA, cna, ebpS, eno, sdrC, sdrE*	30/59			30/30
XIII	*SeC, hla, hlb, icaA, icaB, icaD, icaR, clfA, fnbpA, fnbpB, cidA, fib, cna, ebpS, eno, sdrC, sdrE*	6/12			6/6
XIV	*icaA, icaD, icaR, clfA, cna, eno*	1/2			1/1
XV	*icaA, icaD, icaR, clfA, eno*	4/8			4/4
XVI	*icaA, icaD, icaR, clfA, fnbpA, fib, eno, sdrC*	2/4			2/2
XVII	*icaA, icaD, icaR, clfA, fnbpA, fib, eno*	2/4			2/2
XVIII	*icaA, icaD, icaR, clfA, fnbpA, fnbpB, fib, eno*	1/2			1/1
XIX	*icaA, icaD, icaR, clfA, fnbpA, cidA, fib, eno*	1/2			1/1
XX	*icaA, icaD, icaR, clfA, fnbpA, cidA, fib, eno, sdrC*	1/2			1/1
XXI	*lukED, hla, hlb, icaA, icaR, clfA, cna, eno*	1/2			1/1
XXII	*hla, hlb, icaA, icaD, icaR, clfA, fnbpA, fib, cna, eno*	1/2			1/1
XXIII	*hla, hlb, icaA, icaD, icaR, clfA, fnbpB, cna, eno*	1/2			1/1

1 *icaA* – gene A encoding the intracellular adhesion N-acetylglucosaminyl transferase enzyme synthesising oligomers; *icaB* – gene B encoding the intracellular adhesion deacetylase involved in polysaccharide intercellular adhesin maturation; *icaD* – gene D encoding the intracellular adhesion chaperone protein folding IcaA; *icaR* – gene R encoding the intracellular adhesion transcriptional repressor of the ica operon; *clfA* – gene A encoding clumping factor; *clfB* – gene B encoding clumping factor; *fnbpA* – gene A encoding fibronectin-binding protein; *cidA* – gene A encoding the murein hydrolase regulator; *fib* – gene encoding fibrinogen-binding protein; *cna* – gene encoding collagen-binding protein; *ebpS* – gene encoding *Staphylococcus* elastin-binding protein; *eno* – gene encoding laminin-binding protein; *sdrC* – gene C encoding serine-aspartate repeat protein; *sdrE* – gene E encoding serine-aspartate repeat protein; *fnbpB* – gene B encoding fibronectin-binding protein; *lukED* – genes E and D encoding leukocidins; *hla* – gene a encoding haemolysin; *hlb* – gene β encoding haemolysin; *SeC* – gene C encoding *Staphylococcus* enterotoxin

The analysis of the distribution of virulence gene profiles within individual STs revealed a high degree of differentiation (Table 6). The MRSA and MDR strains mostly belonged to ST 398 (n = 39), among which the dominant virulence gene profiles were XII (n = 30) and XIII (n = 6) (Table 6). These strains were characterised by the same profile of resistance genes (*mecA, mecC, blaZ, ermA, ermB, tetM* and *tetK*). Single isolates belonging to ST 398 represented virulence profiles XXI, XXII and VII. The remaining MDR *S. aureus* belonging to clonal complex 1 (ST 9 and ST 2423) as well as ST 8135 had heterogeneous virulence gene profiles. Extensive virulence repertoires of 13 genes were harboured by VISA strains of ST 133 (of profile IV) and ST 479 (of profile III). A comparably extensive repertoire of 14 was harboured by VISA strains of ST 8139 (of profile I). The presence of the *lukED* gene was detected in one VISA strain (ST 479) (Table 6).

**Table 6.** Distribution of virulence and resistance gene profiles of *Staphylococcus aureus* strains

Host (n)	Clonal complex	Sequence type/n	Methicillin resistance	Multidrug resistance	Vancomycin intermediate resistance	Virulence gene profile	Profile component genes	Resistance genes
	1	9/1	–	+	–	XV	*icaA, icaD, icaR, clfA, eno*	*blaZ*
	1	9/1	–	+	–	XV	*icaA, icaD, icaR, clfA, eno*	*blaZ, tetM*
	1	9/2	–	+	–	XVI	*icaA, icaD, icaR, clfA, fnbpA, fib, eno, sdrC*	*blaZ, tetM*
	1	9/2	–	+	–	XVII	*icaA, icaD, icaR, clfA, fnbpA, fib, eno*	*blaZ, tetM*
	1	9/1	–	+	–	XIX	*icaA, icaD, icaR, clfA, fnbpA, cidA, fib, eno*	*blaZ, tetM*
	1	9/1	–	+	–	XX	*icaA, icaD, icaR, clfA, fnbpA, cidA, fib, eno, sdrC*	*blaZ, tetM*
	1	2423/2	–	+	–	XV	*icaA, icaD, icaR, clfA, eno*	*ermC*
		398/1	+	+	–	XXI	*lukED, hla, hlb, icaA, icaR, clfA, cna, eno*	*mecA, mecC, blaZ, ermB, tetM, tetK, aac*
Pig		398/1	+	+	–	XXII	*hla, hlb, icaA, icaD, icaR, clfA, fnbpA, fib, cna, eno*	*mecA, mecC, blaZ, ermB, tetM, tetK*
		398/1	+	+	–	XXIII	*hla, hlb, icaA, icaD, icaR, clfA, fnbpB, cna, eno*	*mecA, mecC, blaZ, ermB, tetM, tetK*
		398/30	+	+	–	XII	*SeC, hla, hlb, icaA, icaB, icaD, icaR, clfA, fnbpA, fnbpB, cidA, cna, ebpS, eno, sdrC, sdrE*	*mecA, mecC, blaZ, ermA, ermB, tetM, tetK*
		398/6	+	+	–	XIII	*hla, hlb, icaA, icaD, icaR, clfA, fnbpB, cna, eno*	*mecA, mecC, blaZ, ermA, ermB, tetM, tetK*
		8135/1	–	+	–	XIV	*icaA, icaD, icaR, clfA, cna, eno*	*blaZ, ermC, tetM*
		ND/1	–	–	–	XVIII	*icaA, icaD, icaR, clfA, fnbpA, fnbpB, fib, eno*	*blaZ*
		133/1	–	–	+	IV	*icaA, icaB, icaD, icaR, clfA, clfB, fnbpA, cidA, fib, ebpS, eno, sdrC, sdrE*	-
		479/1	–	–	+	VI	*lukED, hla, hlb, icaA, icaB, icaD, icaR, clfA, clfB, fnbpA, cidA, fib, cna, eno*	-
		398/1	+	+	–	VII	*icaA, icaD, icaR, clfA, fnbpA, fib, eno*	*mecA, mecC, blaZ, tetM, tetK*
		8139/1	–	–	+	III	*icaA, icaB, icaD, icaR, clfA, clfB, fnbpA, fnbpB, cidA, fib, ebpS, eno, sdrC*	*blaZ*
		8139/2	–	–	+	I	*icaA, icaB, icaD, icaR, clfA, clfB, fnbpA, cidA, fib, cna, ebpS, eno, sdrC, sdrE*	-
Cow		ND/1	–	–	–	III	*icaA, icaB, icaD, icaR, clfA, clfB, fnbpA, fnbpB, cidA, fib, ebpS, eno, sdrC*	*tetM, tetK*
		ND/13	–	–	–	I	*icaA, icaB, icaD, icaR, clfA, clfB, fnbpA, cidA, fib, cna, ebpS, eno, sdrC, sdrE*	-
		ND/1	–	–	–	V	*icaA, icaB, icaD, icaR, clfA, clfB, fnbpA, cidA, fib, cna, eno*	-
		ND/11	–	–	–	IV	*icaA, icaB, icaD, icaR, clfA, clfB, fnbpA, cidA, fib, ebpS, eno, sdrC, sdrE*	-
		ND/3	–	–	–	II	*icaA, icaB, icaD, icaR, clfA, clfB, fnbpA, cidA, fib, ebpS, eno, sdrC*	-
		ND/1	–	–	–	III	*icaA, icaB, icaD, icaR, clfA, clfB, fnbpA, fnbpB, cidA, fib, ebpS, eno, sdrC*	-
		ND/1	–	–	–	IX	*icaA, icaB, icaD, icaR, clfA, clfB, fnbpA, fnbpB, cidA, fib, cna, ebpS, eno, sdrC, sdrE*	*blaZ, tetM, tetK*
		ND/8	–	–	–	VIII	*icaA, icaB, icaD, icaR, clfA, clfB, fnbpA, cidA, fib, cna, ebpS, eno, sdrE*	-
Wild deer		ND/1	–	–	–	X	*icaA, iaB, icaD, icaR, clfA, clfB, fnbpA, fnbpB, cidA, fib, cna, ebpS, eno, sdrC*	-
		ND/1	–	–	–	IV	*icaA, icaB, icaD, icaR, clfA, clfB, fnbpA, cidA, fib, ebpS, eno, sdrC, sdrE*	-
		ND/1	–	–	–	XI	*icaA, icaB, icaD, icaR, clfA, clfB, fnbpA, cidA,fib, cna, ebpS, eno*	-

1 ND – not determined; *icaA* – gene A encoding the intracellular adhesion N-acetylglucosaminyl transferase enzyme synthesising oligomers; *icaD* – gene D encoding the intracellular adhesion chaperone protein folding IcaA; *icaR* – gene R encoding the intracellular adhesion transcriptional repressor of the ica operon; *clfA* – gene A encoding clumping factor; *eno* – gene encoding laminin-binding protein; *fnbpA* – gene A encoding fibronectin-binding protein; *fib* – gene encoding fibrinogen-binding protein; *sdrC* – gene C encoding serine-aspartate repeat protein; *cidA* – gene A encoding the murein hydrolase regulator; *lukED* – genes E and D encoding leukocidins; *hla* – gene a encoding haemolysin; *hlb* – gene β encoding haemolysin; *cna* – gene encoding collagen-binding protein; *fnbpB* – gene B encoding fibronectin-binding protein; *clfB* – gene B encoding clumping factor; *SeC* – gene C encoding *Staphylococcus* enterotoxin; *sdrE* – gene E encoding serine-aspartate repeat protein; *icaB* – gene B encoding the intracellular adhesion deacetylase involved in polysaccharide intercellular adhesin maturation; *ebpS* – gene encoding *Staphylococcus* elastin-binding protein; *blaZ* – gene encoding β-lactamase Z; *tetM* – gene M encoding a ribosomal protection protein for tetracycline resistance; *ermC* – gene C encoding a ribosomal methyltransferase for macrolide, lincosamide and streptogramin B resistance; *mecA* – gene A encoding a modified penicillin-binding protein; *mecC* – gene C encoding a modified penicillin-binding protein; *ermB* – gene B encoding a ribosomal methyltransferase for macrolide, lincosamide and streptogramin B resistance; *tetK* – gene K encoding a tetracycline efflux pump; *aac* – bifunctional aac(6')-Ie/aph(2")-Ia gene encoding acetyltransferase and phosphotransferase conferring broad aminoglycoside resistance; *ermA* – gene A encoding a ribosomal methyltransferase for macrolide, lincosamide and streptogramin B resistance

**Fig. 2.**
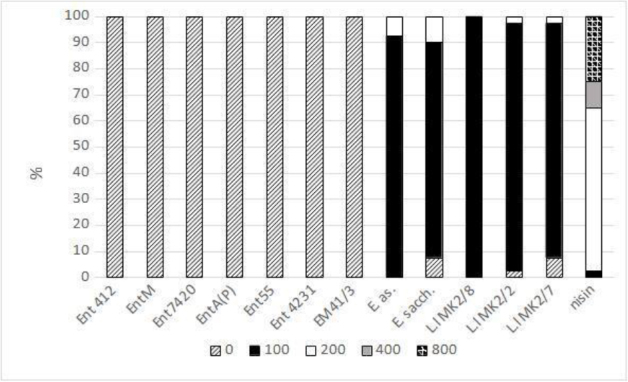
Activity of bacteriocins (arbitrary units/mL) against methicillin-resistant *Staphylococcus aureus* strains. Ent412 – produced by an equine isolate of *Enterococcus faecium*; EntM – produced by an environmental isolate of *E. faecium*; Ent7420 – produced by a leporine isolate of *E. faecium*; EntA(P) – produced by an environmental isolate of *E. faecium*; Ent55 – produced by a poultry isolate of *E. faecium*; Ent 4231 – produced by a vituline isolate of *E. faecium*; EM 41/3 – produced by an equine isolate of *E. mundtii*; E. as. – produced by an equine isolate of *E. asini*; E. sacch. – produced by an equine isolate of *E. saccharolyticus*; L.l MK2 – produced by a caprine isolate of *L. lactis*; nisin – produced by a commercial strain of *L. lactis*. The activity of bacteriocins against some *S. aureus* strains was partially described previously (24, 27)

The analysis of the activity of bacteriocins against MRSA strains showed no inhibitory effect of enterocins produced by *E. faecium* and *E. mundtii* (Fig. 2). The bacteriocins produced by the other *Enterococcus* species (*E. asini* and *E. saccharolyticus*) exerted an inhibitory effect on the growth of the MRSA strains at the level of 100–200 AU/mL. Among the bacteriocins produced by *L. lactis*, nisin showed the highest inhibitory activity (100–800 AU/mL). The activity of the *L. lactis* bacteriocins L.l MK 2/8, L.l MK 2/2 and L.l MK 2/7 against MRSA was in the range of 100–200 AU/mL (Fig. 2).

## Discussion

*Staphylococcus aureus* is a serious medical problem, as it is one of the most dangerous pathogens owing to the presence of numerous virulence factors and the growing phenomenon of resistance, especially to last-resort drugs. The high potential of some strains of these bacteria to produce biofilm, confirmed in this study and observed by other researchers (42), increases their pathogenicity. The noted differences in the biofilm formation ability between strains of *S. aureus* depending on their host may result from different environmental adaptation or survival strategies of these bacteria. Although the small number of tested *S. aureus* isolated from wild deer do not permit the drawing of firm conclusions, the results indicated that these strains were more capable of forming biofilms than those isolated from pigs. This means that this group of hosts, similarly to livestock, should be considered in the future for monitoring the carriage of pathogens dangerous to public health. The need to understand the mechanisms of biofilm formation and regulation is crucial to developing effective treatment strategies for diseases caused by this pathogen (36). This study demonstrates the high carriage of genes encoding MSCRAMMs, namely *eno, clfA, fnbpA, cidA, ebpS, sdrC* and *sdrE*, among *S. aureus* capable of producing biofilm, confirming their important role in the formation of this structure (44). Some of the most frequently detected virulence genes in this study were the components of the *ica* operon, which may also play a key role in biofilm formation. It is interesting that, despite the high frequency of the *icaA, icaD, icaB* and *icaR* genes, the *icaC* gene was not detected in any of the tested strains. This situation may result from the presence of other mechanisms of biofilm formation besides the *ica* operon, which has also been observed by other scientists (33). Moreover, Francois *et al*. (16) reported that mutations in the *ica* operon and the resultant inability to produce PIA had no significant effect on biofilm organisation, which may also indicate the existence of other additional mechanisms of its formation.

The statistically significant differences observed in the occurrence of some genes in isolates from different groups of animals may indicate diverse strategies of biofilm formation depending on the host. Moreover, in their studies, Silva *et al*. (44) showed that the distribution of biofilm-related genes in *S. aureus* strains isolated from different types of human infection was very diverse, and theorised that a particular type of infection may determine a particular pathway of biofilm formation or the absence of its formation. The complexity of the biofilm formation phenomenon found in this study and statistically confirmed in the emergence of an association with strong biofilm formation only for the *clfB* and *fib* genes is consistent with the results reported by Kot *et al*. (23). These authors also reported a high rate of detection of clumping-factor B and fibrinogen-binding protein genes in isolates which formed biofilm.

The multifaceted phenomenon of *S. aureus* virulence has not been fully elucidated. The diversity of virulence profiles of the *S. aureus* strains observed in this study indicates the extremely high pathogenic potential of this species when hosted by farm animals or by wildlife, which could threaten human health. On the other hand, the host-specificity of virulence gene profiles indicates strong adaptation to specific environments. A major engine of variability within *Staphylococcus* species is horizontal gene transfer. It is estimated that up to 22% of the genomic DNA of *S. aureus* may consist of sequences of mobile genetic elements (29). Many virulence genes, including those tested in this study, are located on plasmids, transposons or bacteriophages, where their transfer mainly between strains within the same bacterial species takes place easily. Mobile genetic elements also contribute to a pathogen’s adaptation to new host cells and can have a direct impact on the direction of the pathogen’s genetic evolution, which requires constant monitoring.

The occurrence of certain virulence features combined with resistance to key antibacterial substances requires special attention. Contrary to the significant correlation between MDR and the occurrence of the *SeA* gene observed obtained by Rasmi *et al*. (39), our study shows a strong correlation between multidrug and methicillin resistance and the occurrence of *SeC*. The co-occurrence of genes encoding Hla and Hlb haemolysins and methicillin resistance observed in this study was also confirmed in other publications on strains isolated from humans (12). Considering that in addition to destroying erythrocyte cell membranes, haemolysins are involved in biofilm formation, this observed correlation raises particular concerns and indicates the need to monitor this phenomenon. Another protein also conforms to this co-occurrence pattern: O’Neill *et al*. (34) pointed out the stronger expression of the biofilm-formation protein FnbpB in MRSA strains than in methicillin-susceptible ones, which was also confirmed in our studies.

Thanks to the MLST technique, it is possible to track the spread of *S. aureus* strains, especially MDR variants, MRSA and VISA. The present study aimed to characterise the virulence profiles of MRSA strains and showed the dominance of two similar virulence-gene patterns within the most common ST, which was ST 398. Considering the highly virulent nature of strains of this ST observed in our study (which carried 16 or 17 genes, including those encoding enterotoxins and haemolysins and others associated with biofilm formation) and its noted isolation from farm animals and humans and in the hospital environment, *S. aureus* belonging to ST 398 may pose a serious threat to public health (37). Besides this ST, ST 9 of clonal complex 1 is also a potential concern. It is one of the most frequently recorded sequence types among *S. aureus* isolated from livestock worldwide (50). In our study, MDR strains belonging to this ST were characterised by the presence of PIA locus genes (*icaA, icaD* and *icaR*) and the absence of *lukS*-PV*-lukF*-PV genes, which is consistent with previous studies (50). On the other hand, in contrast to those studies and others conducted by Jiang *et al*. (20), no genes encoding haemolysins were detected among the ST 9 strains in our study. Our results indicated a relatively narrow range of virulence genes within ST 9 compared to other STs, which in the context of previous studies confirmed the plasticity of this ST and may suggest a new path of evolutionary direction (21, 50). Another phenomenon that requires special monitoring is the emergence of new STs as a result of changes in alleles and adaptation to environmental conditions. The new previously unnotified STs (8135 and 8139) observed in our previous studies collected together MDR and VISA strains (48). In this study, we demonstrated their high virulence potential, especially of those in ST 8139 (VISA), which could be agents of a disease with limited treatment options.

Uncontrolled use of antibiotics leading to an increase in drug resistance in bacteria forces the need to look for alternatives in treatment (31). The potential of bacteriocins mainly produced by *E. faecium* and *E. faecalis* against MRSA was observed in our previous study and by other researchers (1, 25). In contrast to these studies and similarly to the results obtained by Park *et al*. (35), in this research, enterocins produced by *E. faecium* did not show activity against MRSA isolated from farm animals. Trivedi *et al*. (47) indicated differences among *Enterococcus* strains in the production of bacteriocins with an anti-MRSA effect, which may explain the lack of such activity noted in the enterocins in our study. There are few studies examining the antimicrobial activity of bacteriocins produced by *E. mundtii* (13, 15). Their results indicate low or no inhibitory activity of the peptides produced by *E. mundtii* against staphylococcal strains, which was also confirmed in this study.

Recently, attention has also been paid to other less known *Enterococcus* species as potential producers of bacteriocins. One such species may be *E. asini*. In our previous work, we demonstrated the high antimicrobial activity of a bacteriocin produced *by E. asini* against Gram-positive bacteria, including various *Staphylococcus* species (27). This study confirmed the high potential of a bacteriocin produced by *E. asini* against MRSA strains, which seems promising but requires further research. Similarly to *E. asini*, another species with potential application may be *E. saccharolyticus*, the bacteriocins of which show activity against MRSA and other *Staphylococcus* species (26).

Bacteria of the *Lactococcus* genus are commonly used in the dairy industry as starter cultures for the production of cheese and yogurt and preservatives through the production of bacteriocins (24). One of the best-known is nisin. In our study, nisin showed the highest inhibitory activity of all the tested bacteriocins against MRSA strains. The potential of nisin to inhibit MRSA growth and biofilm production was previously observed by Silva *et al*. (43). However, their studies indicated that MRSA strains were less inhibited than methicillin-susceptible strains by nisin. The same authors also indicated that individual MRSA strains varied in their susceptibility to nisin, which was also confirmed in our study. This variability may affect the effectiveness of treatment of MRSA infections. Determining the mechanisms of resistance of some bacteria to bacteriocins requires more extensive research.

## Conclusion

The diversity of the occurrence of virulence genes and the ability of *S. aureus* to form biofilms in different hosts indicated the variable adaptive strategies of this pathogen. The co-occurrence of several virulence genes within one *S. aureus* strain resistant to last-resort drugs and affiliated to an ST also infectious to humans indicates livestock and wildlife as potential reservoirs of pathogens dangerous to public health. The demonstrated activity of bacteriocins isolated from *Enterococcus* and *Lactococcus* spp. against MRSA highlights the potential of these compounds as an alternative to classical antibiotic therapy, but this avenue requires further studies.

## Supplementary Material

Supplementary Material Details
