# Supplementary material for: Analysis of the virulence potential, ability to form biofilms and susceptibility to bacteriocins of Staphylococcus aureus strains isolated from livestock and wildlife
Source: J Vet Res. 2026 Feb 5;70(1):21–31. doi: 10.2478/jvetres-2026-0005 (PMC13054752; doi:10.2478/jvetres-2026-0005)
Supplement: Supplementary file 1 — Supplementary Material Details [file jvetres-2026-0005_sm.pdf]

Supplementary Table S1. Characteristics of *Staphylococcus aureus* strains isolated in this study

Host	Clonal complex	Sequence type	n	Resistance profile	Profile resistance genes
Pig	1	9	1	PEN, CLI, TET	*blaZ*
	1	9	3	PEN, CLI, QD, TET	*blaZ*, *tetM*
	1	9	1	PEN, CLI, TET	*blaZ*, *tetM*
	1	9	1	PEN, CLI, TET, ERY	*blaZ*, *tetM*
	1	9	1	PEN, CLI, QD, TET, ERY	*blaZ*, *tetM*
	1	9	1	PEN, CLI, TET, GEN	*blaZ*, *tetM*
	1	2423	1	CLI, QD, ERY	*ermC*
	1	2423	1	CLI, QD, ERY, GEN	*ermC*
		8135	1	PEN, CLI, SXT, QD, TET, ERY, GEN	*blaZ*, *tetM*, *ermC*
		398	1	PEN, CLI, QD, TET, FOX, ERY	*blaZ*, *mecA*, *mecC*, *aac*, *tetM*, *tetK*, *ermB*
		398	1	PEN, CLI, QD, TET, FOX, ERY, GEN	*blaZ*, *mecA*, *mecC*, *tetM*, *tetK*, *ermB*
		398	1	PEN, CLI, SXT, TET, FOX, ERY	*blaZ*, *mecA*, *mecC*, *tetM*, *tetK*, *ermB*
		398	36	PEN, CLI, TET, FOX, ERY	*blaZ*, *mecA*, *mecC*, *tetM*, *tetK*, *ermA ermB*
		ND	1	PEN, CLI	*blaZ*
Cow		398	1	PEN, TET, FOX, CIP, ENR	*blaZ*, *mecA*, *mecC*, *tetM*, *tetK*
		8139	1	VAN	*blaZ*
		8139	2	VAN	-
		133	1	VAN	-
		479	1	VAN	-
		ND	1	TET	*tetM*, *tetK*
		ND	1	CIP	-
		ND	1	PEN	-
		ND	1	CLI, ERY	-
		ND	26	S	-
Wild deer		ND	1	PEN, TET	*blaZ*, *tetM*, *tetK*
		ND	1	PEN	
		ND	1	GEN	
		ND	9	S	

ND – not determined; PEN – penicillin; CLI – clindamycin; TET – tetracycline; QD – quinupristin-dalfopristin; ERY – erythromycin; GEN – gentamicin; SXT – trimethoprim-sulfamethoxazole; FOX – cefoxitin (oxacillin); CIP – ciprofloxacin; ENR – enrofloxacin; VAN – vancomycin; S – susceptible; *blaZ* – gene encoding β-lactamase Z; *tetM* – gene M encoding a ribosomal protection protein for tetracycline resistance; *ermC* – gene C encoding a ribosomal methyltransferase for macrolide, lincosamide and streptogramin B resistance; *mecA* – gene A encoding a modified penicillin-binding protein; *mecC* – gene C encoding a modified penicillin-binding protein; *aac* – bifunctional aac(6')-Ie/aph(2'')-Ia gene encoding acetyltransferase and phosphotransferase conferring broad aminoglycoside resistance; *tetK* – gene K encoding a tetracycline efflux pump; *ermB* – gene B encoding a ribosomal methyltransferase for macrolide, lincosamide and streptogramin B resistance; *ermA* – gene A encoding a ribosomal methyltransferase for macrolide, lincosamide and streptogramin B resistance
